# Genome Sequence of Piezophilic Bacterium *Desulfovibrio profundus* Strain 500-1, Isolated from a Deep Sediment Layer in the Japan Sea

**DOI:** 10.1128/genomeA.01181-17

**Published:** 2017-11-02

**Authors:** Stéphanie Fouteau, Thomas Guerin, Ghislaine Magdelenat, Marie Roumagnac, Manon Bartoli, Bernard Ollivier, Alain Dolla, Valérie Barbe, Nathalie Pradel

**Affiliations:** a Laboratoire de Biologie Moléculaire pour l’Etude des Génomes (LBioMEG) CEA, Institut de Biologie François-Jacob, Genoscope, Evry, France; b Laboratoire de Séquençage (LS) CEA, Institut de Biologie François-Jacob, Genoscope, Evry, France; c Aix Marseille Université, Université de Toulon, CNRS, IRD, MIO, Marseille, France; d Aix Marseille Université, CNRS, LCB, Marseille, France

## Abstract

Piezophilic *Desulfovibrio profundus* strain 500-1 was isolated in the Japan Sea from a sediment layer at 500-m depth under a water column of 1,000 m. Here, we report the genome sequence of this strain, which includes a 4,168,905-bp circular chromosome and two plasmids of 42,836 bp and 6,167 bp.

## GENOME ANNOUNCEMENT

Sulfate-reducing bacteria are widespread in deep-sea environments. *Desulfovibrio profundus*, isolated by Bale and collaborators during the Ocean Drilling Program Leg 128, site 798B (1), is the first piezophilic sulfate-reducing bacterium (SRB) isolated from the sediment layer at 500-m depth under a water column of 1,000 m. The genomes of three other piezophilic *Desulfovibrio* species have been sequenced. They include *D. hydrothermalis*, *D. piezophilus*, and *D. indicus*, isolated from a hydrothermal chimney at 2,600-m depth on the East Paciﬁc Rise (2), wood falls at 1,700-m depth in the Mediterranean Sea (3), and serpentinized peridotite at 3,173-m depth in the Indian Ocean (4), respectively. To gain insight into the genetic traits of the piezophilic *Desulfovibrio* species, we sequenced the genome of *D. profundus* 500-1 and report the complete sequence here.

Genome sequencing was performed mixing Illumina technology and Oxford Nanopore technology (ONT). First, a multiplexed overlapping paired-end library, with 546-bp insert size, was constructed and loaded on an Illumina MiSeq instrument (2 × 300 bp). In parallel, genomic DNA was tagmented to create an ONT library following the manufacturer's recommendations. This library was loaded on MinION R9.4 SpotON flow cells. The Illumina and ONT data, around 270- and 20-fold coverage, respectively, were assembled using SPAdes version 3.6.0 (http://cab.spbu.ru/software/spades/). An optical map of the *D. profundus* 500-1 genome was produced using an Argus system (OpGen). The annotation was performed using the Microscope platform (5).

The whole genome consists of a 4,168,905-bp chromosome and 2 circular plasmids of 42,836 bp and 6,167 bp. The average G+C contents for the DNA are 52.83%, 50.34%, and 45.86%, respectively. A total of 4,008 coding DNA sequences (CDSs) were predicted for the chromosome, as well as 8 pseudogenes, 11 miscellaneous RNAs (misc-RNA), 3 rRNA operons, and 57 tRNA genes. A total of 43 CDSs and 1 misc-RNA were predicted for the largest plasmid, and 7 CDSs were predicted for the second plasmid. Among the NCBI Genomic Reference sequences, the top genome homologous to *D. profundus* 500-1 is that of *D. piezophilus* (GenBank accession no. FO203427 [6]). Reciprocal best BLAST analysis indicated that *D. profundus* shares 2,823 orthologous proteins with *D. piezophilus*. Interestingly, repeated regions account for 10.64% of the genome. By comparison, they represent only 2% in the genome of *D. piezophilus*. The 43 CDSs of the largest plasmid were all classified as unknown, but some of them presented similarities to transposases encoding genes. Among the 7 CDSs of the smallest plasmid, 6 were classified as unknown and 1 as a putative 3-demethylubiquinol 3-*O*-methyltransferase. To our knowledge, the genome of *D. profundus* possesses the largest numbers of CDSs, plasmids, and repeated genomic regions reported for a marine *Desulfovibrio* species to date. It will be of interest to determine the part played by the genomic recombination events in the pressure adaptation capacities of *D. profundus*.

### Accession number(s).

The ﬁnal annotated genome of *D. profundus* is available in GenBank/EMBL under the accession no. LT907975 to LT907977 for the chromosome and the plasmids, respectively.
